# Adiposity and Cardiac Defects: Pathophysiology and Etiology

**DOI:** 10.7759/cureus.34026

**Published:** 2023-01-21

**Authors:** Gaurav Koparkar, Dalia A Biswas

**Affiliations:** 1 Physiology, Jawaharlal Nehru Medical College, Datta Meghe Institute of Higher Education and Research, Wardha, IND

**Keywords:** bmi (body mass index), stroke, hypertension diagnosis, heart failure, adipose tissue, coronary artery disease, cardiovascular disease, obesity-subtype, adiposity, obesity

## Abstract

The worldwide obesity epidemic is well known, with most countries experiencing rises in obesity incidence since the 1980s. Obesity directly contributes to the occurrence of cardiovascular risk factors such as dyslipidemia, type 2 diabetes, hypertension, and sleep problems. Obesity, in addition to other cardiovascular risk factors, contributes to the development of cardiovascular disease (CVD) and cardiovascular disease mortality. Recent research suggests that abdominal obesity, as measured by waist circumference, is a risk factor for CVD that is independent of body mass index. As adipose tissue develops excessively, the individual's heart structure and function undergo a range of adaptations and changes. Obesity is a long-term metabolic disease that is linked to CVD, more hospitalizations, and more deaths. Even in the case of high blood pressure or a persistent structural heart problem, it is clear that when too much fat builds up, the structure and function of the heart change in a number of ways. In addition to its indirect effects, obesity has a number of direct and indirect effects on the cardiovascular system which makes it more likely for people to get sick or die. There may not be a direct link between total body fat and heart rate because the heart rate goes down when body fat percent goes up. High cardiac output in obese people is mostly caused by a rise in stroke volume to meet the metabolic needs of adipose tissue. Cardiomyopathy is caused by a direct effect of obesity on the heart. This is called adipositas cordis. Overweight and obesity can cause or be linked to a number of heart problems, such as coronary artery disease, cardiac arrest, and sudden death.

## Introduction and background

The human body is designed to preserve energy when possible, and the human species has been accustomed to poverty and hunger for a long time. Food is abundant for the first time in history with this generation. Therefore, people eat more by habit and become obese. Excessive food consumption, i.e., eating more than the body requires can lead to obesity. Obesity rates have substantially increased in both sexes and people of all ages, with older people and women seeing proportionately greater prevalence rates [1]. Daily calorie consumption consists of the basal metabolic rate in addition to daily activity and nutritional activity. W/H2 is the formula used to calculate the obesity index, which is used to measure obesity. W stands for weight in kilograms, and H for height in meters. Body mass index (BMI) (kg/m^2^) statistics on obesity are based on the ranges of 18.5-24.9, 25-29.9, and ≥30 for normalcy, overweight, and obesity (Table 1). Men with a BMI above 27.8 kg/m^2^ and women with a BMI over 27.3 kg/m^2^ are considered obese (more than 1.2 times the desirable body weight) [2]. Central obesity may be of the Android or apple kind (abdominal in males) and the Gynecoid variety (around the mammary gland area, hips, and thighs in females) [3].

**Table 1:** Classification of Overweight and Obesity by the Percentage of Body Fat, Body Mass Index (BMI), Waist Circumference, and Associated Diseases Risk From Clinical Guidelines on the Identification, Evaluation, and Treatment of Overweight and Obesity in Adults–The Evidence Report. National Institutes of Health. Obesity Res. 1998;Suppl 2:51S–209S.

	BMI (kg/m^2^)	Men, ≤102 cm; Women, ≤88 cm	Men, >102 cm; Women, >88 cm
Underweight	<18.5	…	…
Normal	18.5-24.9	…	…
Overweight	25.0-29.9	Increased	High
Obesity, class			
I	30.0-34.9	High	Very High
II	35.0-39.9	Very High	Very High
III (Extreme Obesity)	≥40	Extremely High	Extremely High

Obesity in the lower body, which is generated by hyperplasia, the top half of body fat dispersion is mainly induced by hyperplasia (cell enlargement) (rise in the number of adipocytes). There have been discussions regarding genetic predisposition [4]. Significantly elevated cardiovascular risk is linked to obesity. Obesity in the abdomen, considerably if the waist-to-hip ratio is elevated, is more dangerous. Metabolic syndrome (Syndrome-X) is characterized by hyperglycemia, diabetes, dyslipidemia (increased triglyceride and reduced HDL), and obesity [5]. Significant adverse effects of obesity include a more risk of coronary heart disorders, diabetes, high BP, metabolic disease, and a reduced lifespan [6]. A diet low in calories and fat may delay aging and lengthen life.

One in four adults in the United States is obese, while roughly three-quarters of individuals in the country are overweight or obese. Class III adiposity (BMI 40 kg/m^2^) was 7.7 percent of people in the USA in 2016, according to the Centers for Disease Control and Prevention. Adiposity costs the USA an estimated 147 billion to over 210 billion dollars per year in health care expenditures [7]. Over a year, obese workers cost their employers an average of $500 less productivity than their slim counterparts. Being overweight or obese can lead to an elevated risk of circulatory system disease and an increased prevalence and severity of cardiovascular disease (CVD) [8]. The significant reasons are poor dietary practices (overconsumption of high-calorie meals) and inactivity.

In literature search, it has been found that obesity directly contributes to the occurrence of CVD and the increase in morbidity. This article highlights the pathophysiology related to CVD. The present review discusses the negative effects of obesity on CV health manifesting as accelerated progression of atherosclerosis, higher rates of ventricular remodeling, and a higher risk of associated diseases, including stroke, myocardial infarction, and heart failure. The most effective therapies for reversing CVD risk factors associated with obesity have been dietary changes with exercise, especially through structured exercise programs, such as cardiac rehabilitation.

Methodology

**Figure 1:**
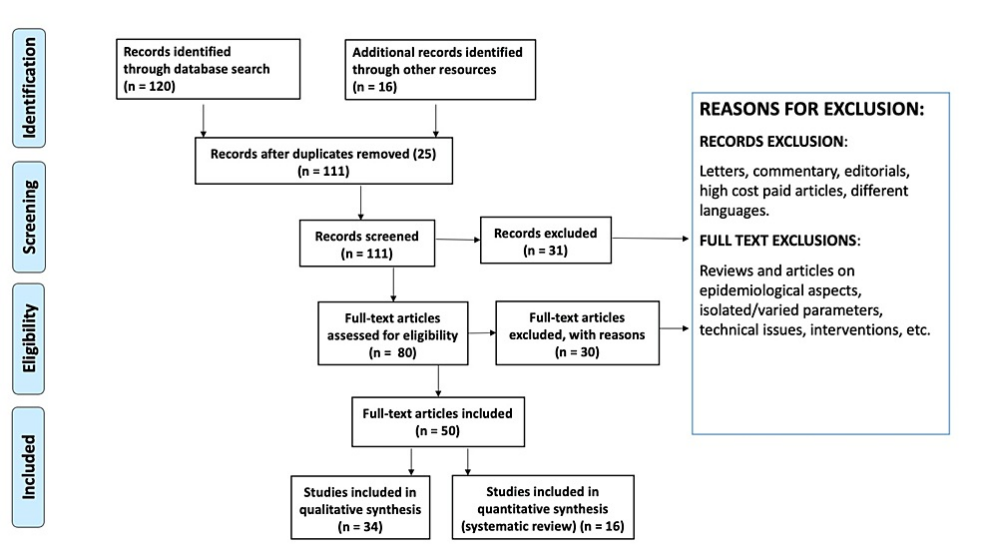
Review of Literature by the PRISMA Method PRISMA: Preferred Reporting Items for Systematic Reviews and Meta-Analyses

We have researched various articles on PubMed Central, MEDLINE, the Cochrane Library Web of Science, and Google Scholar. Search strategies included MeSH words like "adiposity" (AND, OR, #), "cardiovascular diseases" (AND, OR, #), "adipose tissue" (AND, OR, #), "BMI" (AND, OR, #), "adipositas cordis" (AND, OR, #), and "hypertension" (AND, OR, #). To screen the articles, we initially used the abstract and then moved on to full-text examination. Figure 1 shows the flow of study selection according to the Preferred Reporting Items for Systematic Reviews and Meta-Analyses (PRISMA) method.

## Review

Obesity-related circulatory system pathophysiology

Although blood flow to fat tissue is generally between two and three ml/100-g-min at rest, it can surge to ten times that amount after you've had a meal. However, perfusion per unit mass decreases as obesity rates grow [9]. However, circulation per unit mass decreases as obesity rates increase. There may not be a direct correlation between total body fat and cardiac output because cardiac output drops from 2.35 milliliters per minute to 1.50 milliliters per minute whenever body fat percent increases from 20 percent to 36 percent [10].

In obese individuals, there may be a high cardiac output which is caused chiefly by a rise in stroke volume to fulfil the metabolic requirement of adipose tissue. To compensate for the high venous return, the left ventricle enlarges, resulting in eccentric hypertrophy that retains the everyday wall stress. In addition to an enlarged left atrium, obesity is related to a rise in blood volume and venule return. Later, additional issues, such as left ventricular enlargement and diastolic abnormality, may contribute to the enlargement of the left atrium [11].

Body mass was shown to be more closely associated with cardiac output and the mass of the left ventricle as compared to changes in blood volume brought on by obesity in the Strong Heart Study group. At rest, the left ventricle filling pressure rises as predicted, but during exercise, it commonly exceeds 20 mm Hg [12]. The left ventricle experiences eccentric hypertrophy. At first, hyperkinetic systole and left ventricle diastolic malfunction coexist, but if adiposity persists for a longer time, the diastolic dysfunction worsens, and systolic dysfunction subsequently appears [13]. The direct impact of obesity on the heart, known as adipositas cordis, results in cardiomyopathy in obese people. In the beginning, the rise in the heart's fat content results from a metaplastic phenomenon rather than an infiltrative one. Many cardiac components, including the sinoatrial node, atrioventricular (AV) node, a right bundle of his branch, and the muscle surrounding the atrioventricular ring, are replaced by fat cells. Conduction abnormalities such as AV block, sinoatrial block, and bundle branch block can occasionally result from these [14].Adipose tissue bands that are not regular may eventually split, leading to myocardial cell atrophy under strain. Additionally, these adipose cells have the potential to release locally active chemicals like adipokines, which might harm the myocardial cells next to them [15]. Cell malfunction brought on by lipid toxicity can also be caused directly by triglyceride accumulation in no-fat-containing cells such as myocytes.

Obesity as a possible cause of metabolic/genetic cardiovascular disorder

The number of persons who have Metabolic Syndrome (Met S) has skyrocketed across the world. There were several conceptions of the Met S, though now it is a well-defined syndrome [16]. Diabetes and cardiac disorders risk correlate with Syndrome X as it increases the risk of coronary artery diseases (CAD) [17]. Although abdominal obesity has become a universal risk factor for circulatory system disease, the anticipated number of life years lost owing to adiposity differs by sex and race [18].

Currently, more than 41 genomic locations have been recognized as possible connections to the emergence of obesity under favorable conditions [4]. In the absence of co-morbidities, a person with excess adipose tissue has several abridged heart shape and function versions, in addition to a modified metabolic profile. Overall, due to its effects on the circulatory system, overweight and obesity predispose to or are linked to a number of cardiac problems, including CAD, cardiac arrest, and sudden death [19].

Adipositas cordis

It is a myocardiopathy of adiposity. Rather than being caused by an infiltrative process, the fatty heart is presumably caused by a metaplastic phenomenon. Fat can slowly build up between muscle fibers in cell cords, and it can also cause myocyte degeneration, which can lead to heart dysfunction. Small irregular bands and clusters of adipose tissue may be seen here, which may be the result of the intervening fat causing pressure-induced atrophy in the myocardial cells.

The lipid toxicity of the heart caused by free fatty acids, which can lead to the death of lipid-rich cells like cardiomyocytes, is another theory for pathophysiology of obesity-related cardiomyopathy [20]. Obesity may raise the risk of heart failure through a number of processes (elevated cardiac output, higher total blood volume, left ventricular diastolic dysfunction and hypertrophy, and adipose tissue). Because lower leg edema and dyspnea with exercise are frequently nonspecific symptoms of the cardiac disorder in adiposity, it could be challenging to clinically evaluate an overweight person due to a number of constraints imposed by the subject's morphology [21].

Congestive heart failure

An elevation in blood volume, increased cardiac output, hypertrophy of the left ventricle, and malfunction of the left ventricle diastolic are all key obesity-related heart failure symptoms [22]. Along with direct consequences, other often comorbid diseases, including diabetes, hypertension, and coronary artery disease, can also have indirect effects. For a variety of reasons, clinical evaluation of obese individuals for heart failure is sometimes challenging. Leg edema and exertional dyspnea can happen even in the absence of congestive heart failure [23]. When they are lying down, their stomach contents may press against their diaphragm, resulting in orthopnea. Physical examinations are challenging since it is challenging to view neck veins, hear heartbeats and breath noises, and palpate the liver, even if it gets more prominent due to heart failure.

Heart failure occurred in 496 of the 5880 participants (mean age around 55 years, among which half of them were women) in the Framingham heart study after 14 years of follow-up [24]. Each increment of 1 above 30 on the BMI was calculated to increase the probability of heart failure by five percent in men and seven percent in females after optimizing for known risk factors. The hazard ratio for obese women was 2.12 (95% confidence interval (CI): 1.51-2.97), and for males, it was 1.9 (95% CI: 1.3-.79) [25].

Compared to 54 percent of individuals with normal weight, 42 percent of obese heart failure patients had an ejection fraction of less than 40 percent. As a result, individuals with diastolic heart failure are more prevalent among fat people. Obesity alone is the cause of 11 percent of heart failure in males and 14 percent in females [25].

Arrhythmias

Sudden cardiac death, as well as irregular heartbeats, is more likely to occur in obese individuals. A frequent cause of death for obese people is cardiac arrhythmias. Men and women who were obese had a fourfold increase in the risk of sudden cardiac death, according to the Framingham Heart Study [26].

In different research, the prevalence of severe adiposity was six times more in those aged 25 between 34 and twelve times higher in people aged thirty-five to forty-four, respectively [27]. About 30% of the obese people with glucose intolerance who took part in the NHANES III trial had an abnormally long corrected QT interval. According to studies, 2% and 8% of obese persons exhibited QTc intervals that were greater than 0.46 seconds and larger than 0.44 seconds, respectively. A QTc interval greater than 0.42 seconds was related to an increased risk of death among "healthy" obese persons who were monitored for 15 years. There was an increase in the degree of cardiac repolarization irregularity in obese patients as well as in those with normal weight QT dispersion. For each EKG lead, it compares the greatest and minimum intervals of the QT interval [28]. QTc interval and QT dispersion are influenced by changes in the sympathetic-vagal balance. Higher amounts of catecholamines are found in obese persons [29].

In individuals with myocardial infarction, irregular ventricular beat and long-chain trans fatty acids levels are linked. Weight gain affects the autonomic system in a variety of ways. With a 10% increase in body weight, the parasympathetic tone decreases, and the heart rate rises. Heart rate decreases as weight is reduced, on the other hand. The variability of heart rate rises noticeably when a person loses 10 percent of their body weight. Both a greater resting heart rate and a lesser circadian rhythm are risk factors for death, regardless of ejection fraction [30].

The Harvard public health nurse's research [31], the Manitoba study [32], and the Framingham heart study [33] all found that obesity is a standalone predictor of coronary heart disorder. Patients in the Framingham cohort were monitored for an average of 26 years, ranging in age from 28 to 62. The risk of coronary artery disorder was double among males under 50 who were overweight compared to those who were slimmer. After correcting for other important cardiovascular risk factors, the risk was shown to be increased by 2.5-fold in obese women of approximately the same age [33].

Moreover, obese patients who have coronary artery bypass have a higher incidence of poor outcomes. They are more prone to sternum infections, saphenous vein collection site infections, and thromboembolism after surgery [27]. Additionally, atrial arrhythmias are becoming more prevalent. However, neither mortality nor cerebrovascular events following surgery rose significantly.

Atrial fibrillation

According to estimates, obesity may be responsible for 60% of recent population growth and one-fifth of cases of atrial fibrillation (AF) [34,35]. In later life, incident AF is substantially linked with weight increase and a higher midlife BMI [36]. There is a 29% increased risk of incident AF for every five-unit increase in BMI. Furthermore, when body fat distribution is taken into account, these numbers can understate the effect of adiposity. In addition, postoperative AF increases by 10% and postablation AF increases by 13% for every five-unit increase in BMI [36]. The likelihood of the condition progressing from paroxysmal to permanent AF has also been shown to increase with obesity, with class 2 obesity (BMI 35.0 to 39.9 kg/m^2^) being associated with an 87% increase in risk and a BMI in the range of 30 to 34.9 kg/m^2^ being associated with a 54% increase in likelihood [37]. Short-term weight gain results in progressive remodeling of the atria, including increased fibrous tissue deposition, increased expression of endothelin receptors, and abnormalities in atrial conduction, which in turn resulted in increased AF inducibility, according to experimental studies in the ovine mode [38]. These conclusions were expanded upon in a later chronic obesity ovine model, which also described a novel element of the substrate for AF. This investigation showed that the quantities of pericardial fat had significantly increased [39]. Epicardial fat was seen entering the atrial myocardium in histological samples taken from areas close to pericardial fat depots, which could lead to voltage anomalies, conduction obstruction, and increased AF vulnerability. Clinical evidence also shows how obesity and epicardial fat contribute to the development of AF [40]. Early research showed that patients with obesity undergoing electrophysiological tests were considerably more likely to have higher left atrial pressure and volumes than people with normal weight [41]. Additionally, considerable left atrial remodeling and decreased contractility were seen in obese people. After accounting for prevalent cardiovascular risk factors such as diabetes, hypertension, and sleep apnea, these characteristics were still significant. Recent studies used cardiac magnetic resonance imaging and electroanatomic mapping of the left atrium to examine a larger cohort of patients receiving AF ablation. According to this study, obese patients had much greater atrial remodeling, including low-voltage regions, slowed conduction, and higher ECG fractionation [39]. Greater epicardial fat depots were found in areas with more noticeable alterations, demonstrating the contribution of epicardial fat to the promotion of AF. Epicardial adipose tissue has come to light as a significant proarrhythmic substrate which may help explain why obesity increases the risk of AF [42]. The correlations between AF and epicardial fat are stronger than those between AF and measures of total and abdominal obesity, suggesting that adiposity may have a higher impact than previously thought when measured by BMI alone [43]. Potential paracrine signaling is supported by the epicardial adipose tissue's physical closeness to the atrial myocardium [44]. Adiposity may result in a susceptible electrophysiological substrate in the atria through a variety of mechanisms, including fatty infiltration, adipokine-mediated fibrosis, and inflammation [45].

Hypertension

When it comes to hypertension, males with BMI lower than 25 have a 15% prevalence, whereas women who have a BMI of 30 or above have a 15% prevalence and 38% prevalence, respectively. As a result of high blood flow to adipose tissue, obesity raises the heart's output, resulting in a rise in blood pressure. We should expect lower peripheral vascular resistance in overweight patients because of the more extensive cross-sectional area of the artery bed. Considering the larger cross-sectional area of an obese person's arterial bed, we should predict low systemic vascular resistance [46]. When blood pressure is normal or even high, it is more prone to develop into hypertension. The sympathetic nervous system is overactive, and hyperinsulinemia, insulin resistance, low-grade inflammation mediated by adipokines, irregular sleep patterns, and obesity all contribute to systemic vascular resistance. Hypertension is increasingly common when fat grows more severe. In the beginning, it could only occur during the day, particularly if sleep apnea coexists.

Pulmonary hypertension affects 15% to 20% of people with obstructive sleep apnea. When there is no daytime hypoxia, this is often minor and only occurs seldom. The range is between 30- and 35-mm Hg. Patients with obstructive sleep apnea are much more likely to be a victim and have a heart attack, strokes, nighttime dysrhythmias, and both left and right heart failure [47].

Stroke

Even when other risk factors such as diabetes, high blood pressure, and high cholesterol are considered, a higher BMI and waist-to-hip ratio are still independent risk factors for stroke [48]. When compared to men with a BMI of 25, those with a BMI of 25 to 30 (8,105 men) and those with a BMI of >30 (1,184 men) had multiple adjusted relative risks of total stroke that were 1.32 (95 percent CI: 1.14-1.54) and 1.91 (95 percent CI: 1.45-2.52), respectively, in the prospective Physician's Health study cohort of 21,414 men [49]. These figures were derived using the Cox proportional hazards model. These groups had respective relative risks of 1.25 (95 percent confidence interval, 0.84-1.88), 1.92 (95 percent confidence interval, 0.94-3.93), and 1.87 (95 percent confidence interval, 1.38-2.54) for hemorrhagic and ischemic strokes, respectively [50].

The multiple potential risks of ischemia managed to increase by four percent and the incidence of hemorrhagic stroke by six percent with each unit rise in BMI score. Uncertainty surrounds the underlying processes through which increased BMI increases stroke risk, regardless of established risk factors like diabetes and hypertension. As a result of obesity's prothrombotic and inflammatory states, the higher amounts of C-reactive proteins, as well as lymphokines, may play a role in thrombotic events [50].

## Conclusions

From the article, we can conclude that obesity and excess weight are linked to an increased risk of CVD. This is a result of obesity, on the one hand, and accompanying medical issues, on the other hand (hypertension, diabetes, insulin resistance, and sleep apnea syndrome). In the event of pre-existing cardiovascular illness, the death rate of overweight and obese patients is frequently lower than that of people of normal body weight, a phenomenon known as the "obesity paradox." Given the increased cardiovascular risk, regular cardiology screening and management of asymptomatic obese patients are critical for the early detection and treatment of subclinical medical issues.

## References

[1] (2000). Obesity: preventing and managing the global epidemic. Report of a WHO consultation. World Health Organ Tech Rep Ser.

[2] Shah NR, Braverman ER (2012). Measuring adiposity in patients: the utility of body mass index (BMI), percent body fat, and leptin. PLoS One.

[3] Løvsletten O, Jacobsen BK, Grimsgaard S (2020). Prevalence of general and abdominal obesity in 2015-2016 and 8-year longitudinal weight and waist circumference changes in adults and elderly: the Tromsø Study. BMJ Open.

[4] Snyder EE, Walts B, Pérusse L, Chagnon YC, Weisnagel SJ, Rankinen T, Bouchard C (2004). The human obesity gene map: the 2003 update. Obes Res.

[5] Grundy SM, Hansen B, Smith SC Jr, Cleeman JI, Kahn RA (2004). Clinical management of metabolic syndrome: report of the American Heart Association/National Heart, Lung, and Blood Institute/American Diabetes Association conference on scientific issues related to management. Circulation.

[6] Mayoral LP, Andrade GM, Mayoral EP (2020). Obesity subtypes, related biomarkers &amp; heterogeneity. Indian J Med Res.

[7] Hales CM, Fryar CD, Carroll MD, Freedman DS, Ogden CL (2018). Trends in obesity and severe obesity prevalence in US youth and adults by sex and age, 2007-2008 to 2015-2016. JAMA.

[8] Baskin ML, Ard J, Franklin F, Allison DB (2005). Prevalence of obesity in the United States. Obes Rev.

[9] Larsen OA, Lassen NA, Quaade F (1966). Blood flow through human adipose tissue determined with radioactive xenon. Acta Physiol Scand.

[10] Summers LK, Samra JS, Humphreys SM, Morris RJ, Frayn KN (1996). Subcutaneous abdominal adipose tissue blood flow: variation within and between subjects and relationship to obesity. Clin Sci (Lond).

[11] Kaltman AJ, Goldring RM (1976). Role of circulatory congestion in the cardiorespiratory failure of obesity. Am J Med.

[12] Collis T, Devereux RB, Roman MJ (2001). Relations of stroke volume and cardiac output to body composition: the strong heart study. Circulation.

[13] Caballero B (2019). Humans against Obesity: who will win?. Adv Nutr.

[14] Balsaver AM, Morales AR, Whitehouse FW (1967). Fat infiltration of myocardium as a cause of cardiac conduction defect. Am J Cardiol.

[15] Koliaki C, Liatis S, Kokkinos A (2019). Obesity and cardiovascular disease: revisiting an old relationship. Metabolism.

[16] Yusuf S, Vaz M, Pais P (2004). Tackling the challenge of cardiovascular disease burden in developing countries. Am Heart J.

[17] Ginsberg HN, MacCallum PR (2009). The obesity, metabolic syndrome, and type 2 diabetes mellitus pandemic: Part I. Increased cardiovascular disease risk and the importance of atherogenic dyslipidemia in persons with the metabolic syndrome and type 2 diabetes mellitus. J Cardiometab Syndr.

[18] Salim Y, Hawken S, Ôunpuu S (2004). Effect of potentially modifiable risk factors associated with myocardial infarction in 52 countries (the INTERHEART study): case-control study. Lancet.

[19] Poirier P, Martin J, Marceau P, Biron S, Marceau S (2004). Impact of bariatric surgery on cardiac structure, function and clinical manifestations in morbid obesity. Expert Rev Cardiovasc Ther.

[20] Dervan JP, Ilercil A, Kane PB, Anagnostopoulos C (1991). Fatty infiltration: another restrictive cardiomyopathic pattern. Cathet Cardiovasc Diagn.

[21] Karason K, Lindroos AK, Stenlöf K, Sjöström L (2000). Relief of cardiorespiratory symptoms and increased physical activity after surgically induced weight loss: results from the Swedish Obese subjects study. Arch Intern Med.

[22] Poirier P, Giles TD, Bray GA, Hong Y, Stern JS, Pi-Sunyer FX, Eckel RH (2006). Obesity and cardiovascular disease: pathophysiology, evaluation, and effect of weight loss: an update of the 1997 American Heart Association Scientific Statement on Obesity and Heart Disease from the Obesity Committee of the Council on Nutrition, Physical Activity, and Metabolism. Circulation.

[23] Karason K, Girerd N, Andersson-Asssarsson J (2022). Heart failure in obesity: insights from proteomics in patients treated with or without weight-loss surgery. Int J Obes (Lond).

[24] Johnson AL, Cornoni JC, Cassel JC, Tyroler HA, Heyden S, Hames CG (1975). Influence of race, sex and weight on blood pressure behavior in young adults. Am J Cardiol.

[25] Kenchaiah S, Evans JC, Levy D (2002). Obesity and the risk of heart failure. N Engl J Med.

[26] Kannel WB, Plehn JF, Cupples LA (1988). Cardiac failure and sudden death in the Framingham Study. Am Heart J.

[27] Drenick EJ, Bale GS, Seltzer F, Johnson DG (1980). Excessive mortality and causes of death in morbidly obese men. JAMA.

[28] Pontiroli AE, Pizzocri P, Saibene A, Girola A, Koprivec D, Fragasso G (2004). Left ventricular hypertrophy and QT interval in obesity and in hypertension: effects of weight loss and of normalisation of blood pressure. Int J Obes Relat Metab Disord.

[29] Esposito K, Nicoletti G, Marzano S (2002). Autonomic dysfunction associates with prolongation of QT intervals and blunted night BP in obese women with visceral obesity. J Endocrinol Invest.

[30] Lalani AP, Kanna B, John J, Ferrick KJ, Huber MS, Shapiro LE (2000). Abnormal signal-averaged electrocardiogram (SAECG) in obesity. Obes Res.

[31] Manson JE, Colditz GA, Stampfer MJ (1990). A prospective study of obesity and risk of coronary heart disease in women. N Engl J Med.

[32] Rabkin SW, Mathewson FA, Hsu PH (1977). Relation of body weight to development of ischemic heart disease in a cohort of young North American men after a 26 year observation period: the Manitoba Study. Am J Cardiol.

[33] Hubert HB, Feinleib M, McNamara PM, Castelli WP (1983). Obesity as an independent risk factor for cardiovascular disease: a 26-year follow-up of participants in the Framingham Heart Study. Circulation.

[34] Schnabel RB, Yin X, Gona P (2015). 50 year trends in atrial fibrillation prevalence, incidence, risk factors, and mortality in the Framingham Heart Study: a cohort study. Lancet.

[35] Huxley RR, Lopez FL, Folsom AR (2011). Absolute and attributable risks of atrial fibrillation in relation to optimal and borderline risk factors: the Atherosclerosis Risk in Communities (ARIC) study. Circulation.

[36] Wong CX, Sullivan T, Sun MT (2015). Obesity and the risk of incident, post-operative, and post-ablation atrial fibrillation: a meta-analysis of 626,603 individuals in 51 studies. JACC Clin Electrophysiol.

[37] Tsang TS, Barnes ME, Miyasaka Y (2008). Obesity as a risk factor for the progression of paroxysmal to permanent atrial fibrillation: a longitudinal cohort study of 21 years. Eur Heart J.

[38] Abed HS, Samuel CS, Lau DH (2013). Obesity results in progressive atrial structural and electrical remodeling: implications for atrial fibrillation. Heart Rhythm.

[39] Mahajan R, Nelson A, Pathak RK (2018). Electroanatomical remodeling of the atria in obesity: impact of adjacent epicardial fat. JACC Clin Electrophysiol.

[40] Mahajan R, Lau DH, Brooks AG (2015). Electrophysiological, electroanatomical, and structural remodeling of the atria as consequences of sustained obesity. J Am Coll Cardiol.

[41] Munger TM, Dong YX, Masaki M (2012). Electrophysiological and hemodynamic characteristics associated with obesity in patients with atrial fibrillation. J Am Coll Cardiol.

[42] Al Chekakie MO, Welles CC, Metoyer R (2010). Pericardial fat is independently associated with human atrial fibrillation. J Am Coll Cardiol.

[43] Wong CX, Sun MT, Odutayo A (2016). Associations of epicardial, abdominal, and overall adiposity with atrial fibrillation. Circ Arrhythm Electrophysiol.

[44] Hatem SN, Sanders P (2014). Epicardial adipose tissue and atrial fibrillation. Cardiovasc Res.

[45] Lavie CJ, Pandey A, Lau DH, Alpert MA, Sanders P (2017). Obesity and atrial fibrillation prevalence, pathogenesis, and prognosis: effects of weight loss and exercise. J Am Coll Cardiol.

[46] Novi RF, Porta M, Lamberto M, Molinatti GM (1989). Reductions of body weight and blood pressure in obese hypertensive patients treated by diet. A retrospective study. Panminerva Med.

[47] Partinen M, Jamieson A, Guilleminault C (1988). Long-term outcome for obstructive sleep apnea syndrome patients. Mortality. Chest.

[48] Abbott RD, Behrens GR, Sharp DS (1994). Body mass index and thromboembolic stroke in nonsmoking men in older middle age. The Honolulu Heart Program. Stroke.

[49] Folsom AR, Prineas RJ, Kaye SA, Munger RG (1990). Incidence of hypertension and stroke in relation to body fat distribution and other risk factors in older women. Stroke.

[50] Kurth T, Gaziano JM, Berger K (2002). Body mass index and the risk of stroke in men. Arch Intern Med.

